# Epidemic Diseases Present in All Ireland on the Night of March 30th, 1851

**Published:** 1855-06

**Authors:** 


### EPIDEMIC DISEASES PRESENT IN ALL IRELAND ON THE NIGHT OF MARCH 30TH, 1851.

The history of the cases of disease present in Ireland at the moment of taking of the census on the 30th of March 1851, is, without doubt, one of the most curious historical documents of disease that has ever been published; and the profession and public owe to Mr. Wilde, of Dublin, a debt of deep gratitude. We extract from the Report of the Census Commissioners the following valuable statement on the number of cases of epidemic disease present on the 30th of March.

*Total of Epidemic Diseases.*— The number of persons labouring under epidemic diseases far out-numbered those from all other causes, amounting to 34,998, or 1 in 3 of the total sick. Great variety existed in the provincial summaries for this class of disease, in proportion to the population; thus, in Leinster and Connaught, one person in every 209 was returned as sick from some of the causes specified under the class of epidemic disease: in Munster, as many as 1 in 106; whereas, the proportion in Ulster was only 1 in 432.

In examining more minutely into the distribution of the epidemic class of disease, we find a remarkable difference in particular localities, it being greatest in the city of Kilkenny and the counties of Clare and Kerry, the city of Waterford and the town of Galway, in which localities the proportion varied from 1 in 55 to 1 in 94 of the population; and least in the counties of Antrim, Down, Armagh, Donegal and Dublin, and also Belfast town, shewing, in the former instance, the effects of poverty and destitution in the production and maintenance of epidemic diseases, and in the latter, those of comfort, industry, and cleanliness in maintaining a comparative immunity from diseases of an epidemic or contagious character.

Taking up the class of epidemic diseases in detail, we find that the items exhibiting the greatest number are fever, dysentery and diarrhœa taken together, and ophthalmia and influenza. The total number of persons who laboured under fever on the night of the 30th of March 1851, was 13,777, or 1 in 8 of the entire number from all cases. Compared with the population, it prevailed principally in those localites which we have already specified as exhibiting the greatest amount of epidemic disease; but its principal habitats were the workhouses and the workhouse hospitals, as many as 7,888 of the whole amount of fever cases being returned from these localities. Dysentery and diarrhœa may be classed together: their united numbers amount to 9,729: they usually follow in the track of fever; the sexes, however, were more nearly equal than in fever cases. The persons labouring under these diseases were, as well as the former, chiefly located in the workhouse. The returns for ophthalmia amounts to 3,883: it prevailed most in the county of Cork, where as many as 1133, or 1 in every 50 of the population, were affected; it was also very rife in Tipperary, and in the county and city of Limerick. The next important disease was influenza, amounting to 3542; the cold east winds of spring, and a partial epidemic of the disease, serving to increase the prevalence of this disorder, at the time of taking the census. Of those diseases which chiefly affect the young, measles was at the time most prevalent, amounting to 1035. The cases of small-pox returned were 888, a very large amount, considering the safeguard afforded by vaccination against the ravages of this pestilence, and the extent of which can only be accounted for by the disease being maintained and propagated in the country districts by inoculation with small-pox virus, as well as the by general neglect of vaccination. Of the entire number of small-pox cases, 363 were returned as sick at their own homes, 9 were in public hospitals, and 516 were in workhouses and workhouse hospitals. The other diseases enumerated in this class appear to be about the ordinary average; 6 persons were affected with glanders, a disease which appears to affect the human subject more frequently than formerly; and, as it is invariably derived from infected animals of the equine species, it is one which should, properly, come under the surveillance of the police.

The seasons exercise considerable influence upon most of the diseases enumerated in the zymotic class, especially those which are of an acknowledged epidemic disposition. Spring, the period at which the inquiry was made, may be taken as affording rather more than the average amount of small-pox, measles, scarlatina, hooping cough, and also of influenza, which chiefly seize upon the young; whereas, fever, though very prevalent in spring, seldom rises to its intensity till summer and autumn; and diarrhœa and dysentery also prevail more at the latter period than at any of the other seasons: ophthalmia, however, generally spreads extensively both in spring and autumn.

The sexes differ most among persons labouring under the tegumentary system, being, in that class, 100 males to 67·77 females; and next in variety of sexes the persons affected with epidemic diseases, being 100 females to 87·64 males: this arises chiefly from the greater number of females who laboured under fever and ophthalmia.

The ages of persons affected with epidemic diseases were as follows:—of the 34,998 sick persons whose ages were returned, 8,251 were under ten years of age; 12,077, above ten and under twenty; and 14,670, twenty and upwards.

---

